# Adverse reactions following COVID-19 vaccine among healthcare professionals working in Ethiopia: a facility-based cross-sectional study

**DOI:** 10.3389/fpubh.2023.1187948

**Published:** 2023-11-02

**Authors:** Adisu Asefa, Nitsuh Derjachew, Abebe Muche Belete, Feredegn Talargia, Daniel Molla Melese, Bekalu Getachew

**Affiliations:** ^1^Department of Biomedical Science, College of Medicine, Debre Berhan University, Debre Berhan, Ethiopia; ^2^Department of Public Health, College of Health Science, Gamby Medical and Business College, Bahir Dar, Ethiopia; ^3^Department of Biomedical Science, College of Medicine, Jimma University, Jimma, Ethiopia

**Keywords:** COVID-19 vaccine, healthcare professionals, vaccine, Oxford AstraZeneca, corona virus

## Abstract

**Background of the study:**

One of the best medical approaches for halting the spread of infectious diseases is vaccination. During the COVID-19 pandemic, healthcare workers (HCWs) were a high-risk population. Due to their susceptibility in terms of their working environment, front-line healthcare personnel should receive vaccinations before others.

**Objective:**

The purpose of this study was to assess the adverse reactions to COVID-19 vaccines among Ethiopian healthcare professionals in 2022.

**Methods:**

A facility-based cross-sectional study design was conducted in Addis Ababa Health Facilities, Ethiopia. A total of 290 health professionals who were vaccinated during the study period were involved. Data entry was done by Epidata (version 3.1) and analyzed using SPSS software version 26. Bivariable analysis was conducted and a *p* value of less than 0.25 was selected for further multivariable analysis. A *p* value of 0.05 was considered statistically significant at a 95% confidence level.

**Results:**

A total of 277 study participants were successfully involved in the study, yielding a response rate of 95.5%. The study participants comprised 123 (44.4%) women and 154 (55.6%) men. The majority of them (202, 72.9%) had received the Oxford AstraZeneca vaccine. Among the 277 study participants, 142 (51.3%) had developed adverse reactions associated with vaccination. Of these, 81 (29.2%) had moderate adverse reactions. Only 2 (0.7%) had developed adverse reactions that led to hospitalization. The most reported short-term adverse reactions were injection site pain (151, 54.5%), headache (114, 41.2%), fever (104, 37.5%), fatigability and tiredness (94, 33.9%), chills (92, 33.2%), muscle pain (79, 28.5%), and decreased sleep quality (34, 12.3%). The multivariable logistic regression showed that the odds of having an adverse reaction were 1.501 times higher among women than men (AOR = 1.501, 95% CI [1.08, 2.754]).

**Conclusion and recommendations:**

This study revealed that adverse effects following the COVID-19 vaccine were moderate in magnitude and minimal in severity. This study showed that adverse reactions that led to hospitalization were rare. Based on the findings of this study, it is recommended that national, multicenter, prospective, and randomized studies be conducted to assess the independent association of each vaccine.

## Introduction

One of the best medical approaches for halting the spread of infectious diseases is vaccination. To protect communities from COVID-19 and prevent further economic hardship, safe and effective SARS-CoV-2 vaccinations are required (1).

A 94.1% efficacy of the SARS-CoV-2 vaccine (mRNA-1273) has been confirmed, and the first human clinical study of the vaccine began in March 2020 in the United States. However, the SARS-CoV-2 vaccine’s global uptake is still insufficient for herd immunity (2).

Healthcare workers (HCWs) are a high-risk population during the COVID-19 pandemic. This subpopulation has a 9–11 times higher infection risk than the general population (3). In China, a total of 1,433 healthcare workers (HCWs) received vaccinations, and 135 of them reported adverse reactions (9.4%) (4).

According to a study done in India, 98.2% of people experienced adverse effects following immunization. In this study, generalized weakness, local pain, or swelling at the injection site were some of the side effects that were frequently experienced after vaccination. In this study, women (67.7%) were more likely than men (32.3%) to experience detrimental impacts when working as healthcare professionals (5).

The Centers for Disease Control and Prevention (CDC) and other studies have shown that symptoms at the injection site (swelling, pain, and redness) and systemic effects (back pain, fatigue, headache, muscle pain, joint pain, chills, fever, and nausea) were connected to post-COVID-19 vaccination (6).

According to a Chinese study, the two most common complaints were weakness (74, 5.2%) and headache/dizziness (58, 4.0%). The most often reported side effects associated with the COVID-19 vaccination include headache, weariness, muscle and joint pain, fever and chills, and soreness at the injection site (7).

According to a study from Nigeria, participants who had previously experienced an adverse reaction to a medication or vaccination were younger (40 years old), had received two doses, and reported experiencing symptoms more frequently. Approximately 71.1% of the 295 vaccine recipients in Nigeria who participated in the trial experienced at least one side effect (8).

Another study conducted on Ghanaian healthcare workers showed that 528 (80.7%) of the participants reported having adverse reactions. The most common adverse effects among Ghanaian healthcare workers were generalized weakness (32.0%), headache (27.3%), and fever (19.1%) (9). According to a study, healthcare workers aged 35–39 and 40–44 had reduced probabilities of adverse reactions compared to those aged 25–29. Analgesics used by medical personnel before immunization reduced the risk of negative reactions (9).

A study done in Togo revealed that out of 1,639 medical professionals, 71.6% of participants reported at least one adverse effect (10). According to a study done in Ethiopia, 510 (75.7%) medical professionals who received the vaccination reported injection site symptoms of pain (65.48%) and discomfort (57.9%) (11).

Since evidence of the adverse effects of all vaccines given in Ethiopia is scarce, this study was conducted to quickly document adverse events to reassure the population. This study was intended to assess adverse reactions following COVID-19 vaccination and their associated factors among healthcare professionals working in Ethiopia.

## Materials and methods

### Study design and setting

A facility-based cross-sectional study was carried out among healthcare professionals working in Addis Ababa Public Health Facilities, Ethiopia from February 10, 2022 to June 10, 2022.

### Sample size determination

According to a previous study conducted in Ethiopia, 75.8% of healthcare professionals who received the Oxford AstraZeneca vaccine reported injection site symptoms of pain and tenderness (11). Based on this assumption, the minimum sample size required for this study was determined using the single population proportion formula.

$$\left(ni=\left(Z\alpha /2\right)2\;p\;\left(1-p\right)/d2\right)$$

Taking *p* = 75.8%, 5% level of precision (d) with a 95% confidence interval, and a 10% non-response rate was added. Since the source population was 4,471, the population correction formula was utilized. The final sample size was = 290.

### Sampling procedures and techniques

From a total of 11 Governmental Hospitals in Addis Ababa, three were selected by simple random sampling technique (lottery method). The selected Governmental Hospitals included St. Paul’s Millennium Medical College, Yekatit 12 Hospital Medical College, and Eka Kotebe General Hospital. Based on the data from the Addis Ababa Health Bureau and the Federal Ministry of Health, the total number of healthcare professionals working in Addis Ababa governmental hospitals was 4,471.

A systematic random sampling technique was employed after using proportional allocation. The sampling fraction was: 4,471/290 = 15. The first sample was selected using a simple random sampling technique. Then, every 15 healthcare professionals were included in the study from each of the governmental hospitals until the calculated sample size was achieved.

### Study variables

#### Dependent variable

Adverse reactions following COVID-19 vaccine.

#### Independent variables

Socio-demographic factors (age, sex, and educational status), behavioral factors (alcohol drinking, cigarette smoking, khat chewing, and drugs used for any other chronic illnesses), and other factors (type and dose of vaccine, presence of chronic illnesses, and COVID-19 result before or after vaccination).

### Data collection tools and procedures

Data were collected using a structured questionnaire adapted from different literature. The questionnaire includes four parts; socio-demographic characteristics, medical history, behavioral factors, and vaccination status. The questionnaire was prepared in the English language and translated into Amharic and then back into English. Five BSc health professionals were recruited to collect the data and two BSc/MSc health professionals supervised the data collection process. Timely supervision was undertaken by the principal investigator during the data collection period.

### Operational definition

Adverse reactions: unintended pharmacologic reactions that occur when medication or vaccine is administered correctly.
Mild adverse reaction: HCPs who stayed at home to rest and who also took painkillers.
Moderate adverse reaction: HCPs who attended health institutions but did not require hospitalization.
Severe adverse reaction: HCPs who were admitted to hospital and received the required health care services.

### Data processing and analysis

The data were entered into EPI data manager version 3.3 and analyzed using IBM SPSS Statistics version 22. Model fitness was also checked using the Hosmer and Lemeshow test. A summary of descriptive statistics was computed for most variables. A binary logistic regression analysis model was utilized. A point estimate of Odds ratio (OR) with a 95% confidence interval (CI) was determined to assess the strength of association between independent and dependent variables. For all statistically significant tests, value of *p* < 0.05 was used as a cut-off point.

## Results

Out of 290 study participants, 277 were successfully involved in the study, yielding a response rate of 95.5%. The study participants comprised 123 (44.4%) women and 154 (55.6%) men. The study participants’ ages ranged from 22 to 54 years, with mean and standard deviations of 31 and ± 6.46 years, respectively. Most of the participants (127, 45.8%) were nurses in the profession. The majority (266, 96%) had no chronic diseases (Table 1).

**Table 1:** Sociodemographic characteristics of the study participants, Addis Ababa, Ethiopia, 2022.

Variables	Frequency (%)
Gender
Men	154 (55.6)
Women	123 (44.4)
Age
<30	172 (62.1)
30–50	101 (36.5)
>50	4 (1.4)
Job category
Doctors	72 (26)
Health officers	56 (20.2)
Nurses	127 (45.8)
Midwives	10 (3.6)
Medical laboratories	12 (4.3)
Suffering from chronic diseases
Yes	11 (4)
No	266 (96)

### COVID-19 vaccination status

**Figure 1:**
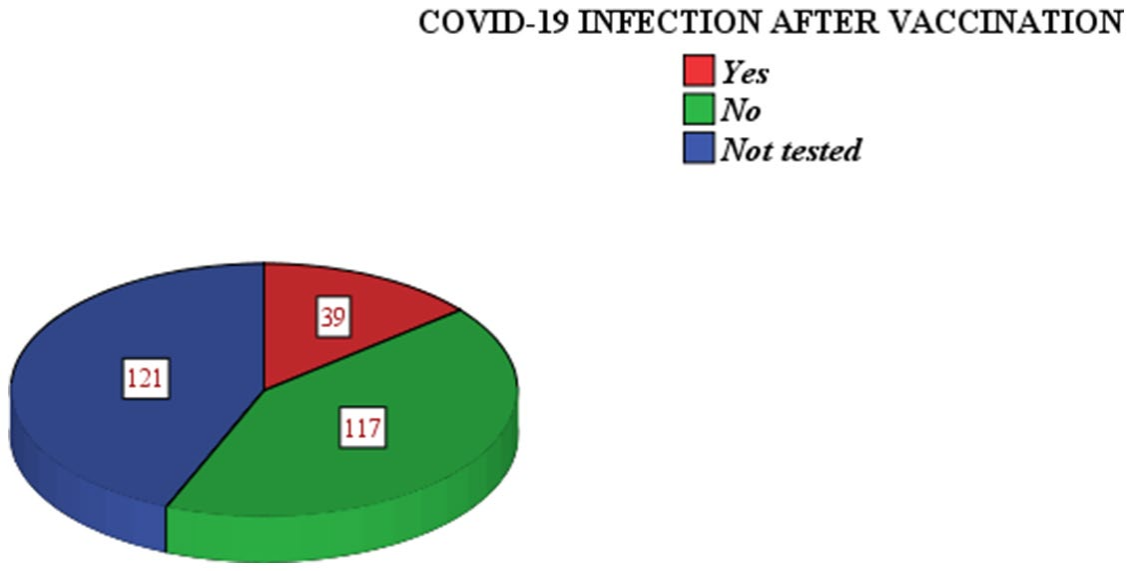
COVID-19 infection after vaccination among health professionals in Addis Ababa, Ethiopia.

The majority (159, 57.4%) of study participants’ previous COVID-19 results were negative. All of the study participants were vaccinated. Among the vaccinated, 39 (14.1%) were infected by the virus (Figure 1).

**Figure 2:**
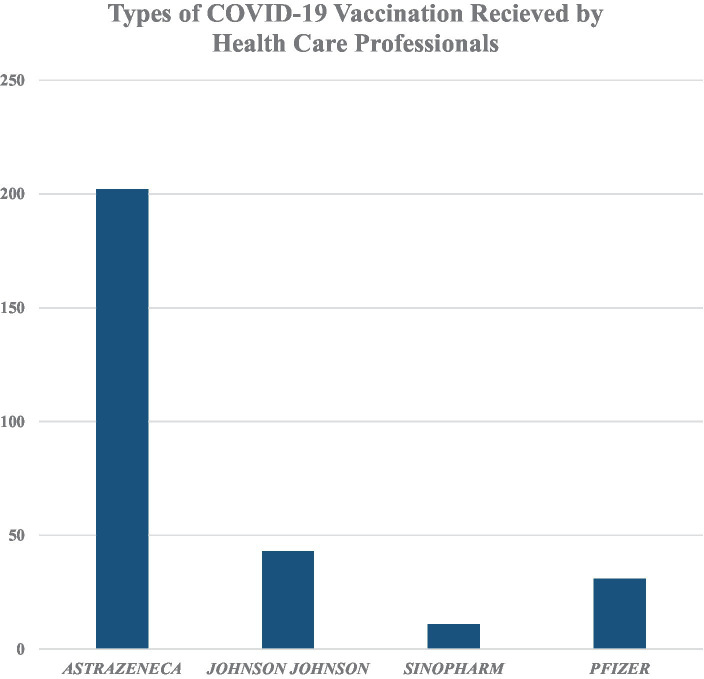
Types of COVID-19 vaccination received by healthcare professionals working in Addis Ababa Public Health Facilities, Ethiopia.

The majority of them (202, 72.9%) had received the Oxford AstraZeneca vaccine. Only 11 (4%) took Sinopharm. Most of the participants (208, 75.1%) received two doses of the COVID-19 vaccine. A total of 249 had no allergies to any types of food or medicines. Only 5 (1.8%) had used substances (Figure 2).

### Prevalence of adverse reactions following COVID-19 vaccine

**Figure 3:**
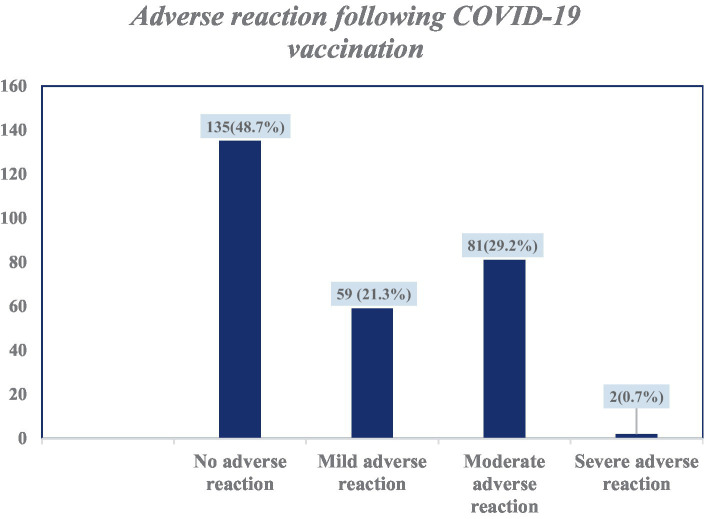
Categories of adverse reactions following COVID-19 vaccination among health care professionals in Addis Ababa, Ethiopia.

Among the 277 study participants, 142 (51.3%) had developed adverse reactions associated with vaccination. Of these, 81 (29.2%) had moderate adverse reactions. Only 2 (0.7%) had developed adverse reactions that led to hospitalization (Figure 3). Among those who had adverse reactions, 70 (25.3%) developed adverse reaction symptoms within 5–8 h of vaccine administration. In the majority (79, 28.5%), the symptoms lasted for 1–3 days. Of the study participants who had received the COVID-19 vaccine, only 2 (0.7%) were diagnosed with thrombosis. Most of the study participants (245, 88.4%) recommended having the COVID-19 vaccine to others.

The most reported short-term adverse reactions were injection site pain 151 (54.5%), headache 114 (41.2%), fever 104 (37.5%), fatigability and tiredness 94 (33.9%), chills 92 (33.2%), muscle pain 79 (28.5%), and decreased sleep quality 34 (12.3%; Table 2).

**Table 2:** Adverse reaction after COVID-19 vaccine administration among health care professionals in Addis Ababa, Ethiopia.

No	Adverse reactions following COVID-19	Frequency
1.	Did you experience any adverse reactions?	
	Yes	142 (51.3%)
	No	135 (48.7%)
2.	Adverse reactions experienced after vaccine administration	
	Injection site pain	151 (54.5%)
	Headache	114 (41.2%)
	Fever	104 (37.5%)
	Fatigability and tiredness	94 (33.9%),
	Chills	92 (33.2%)
	Muscle pain	79 (28.5%)
	Decreased sleep quality	34 (12.3%)
	Nausea and vomiting	24 (8.7%)
	Irritation and skin reaction	19 (6.9%)
	Body sweating	12 (4.3%)
	Runny nose	25 (9%)
	Dyspnea	8 (2.9%)
	Chest pain	14 (5.1%)
	Sore throat	21 (7.6%)
	Cough	28 (10.1%)
3.	How soon the symptoms appeared after injection with a COVID-19 vaccine	
	Up to 4 h	17 (6.1%)
	5–8 h	70 (25.3%)
	9–12 h	41 (14.8%)
	After 24 h	14 (5.1%)
4.	How long the symptoms lasted	
	Less than 1 day	29 (10.5%)
	1–3 days	79 (28.5%)
	4–7 days	34 (12.3%)
	More than 7 days	0
5.	Have you been diagnosed with any types of thrombosis (blood clots)?	
	Yes	2 (1.4%)
	No	140 (98.6%)
6.	Would you recommend the vaccine that you received to others?	
	Yes	245 (88.4%)
	No	32 (11.6%)

### Factors associated with the occurrence of adverse reactions following COVID-19

All sociodemographic characteristics were entered into bivariate logistic regression. Age group (*p* value = 0.23), gender (*p* value = 0.017), job category (*p* value = 0.031), and underlying chronic diseases (*p* value = 0.320). Based on a binary regression result, the odds of having adverse reactions were 1.799 times higher among women than men (COR = 1.799, 95% CI [1.13, 2.906]). The odds of adverse reactions were 66% less likely among study participants who had received two doses of the COVID-19 vaccine than among those who had received it once (COR = 0.337, 95% CI [0.117, 0.975]).

There was no statistically significant association with adverse reactions related to the specific types of COVID-19 vaccine [Oxford AstraZeneca (COR = 1.385, 95% CI [0.814, 2.359]), Johnson & Johnson (COR = 0.90, 95% CI [0.469, 1.727]), Sinopharm (COR = 0.00), and Pfizer (COR = 2.169, 95% CI [0.981, 4.797])].

Multivariable logistic regression analysis has shown that the odds of having adverse reactions were 1.501 times higher among women than men (AOR = 1.501, 95% CI [1.08, 2.754]; Table 3).

**Table 3:** Factors associated with the occurrence of adverse reactions among healthcare professionals in Addis Ababa, Ethiopia.

Variables	*COVID-19 adverse reaction*				
	Yes	No	COR (CI)	AOR (CI)	*p* value
Gender
Men	69 (44.8)	85 (55.2)	1	1	0.01*
Women	73 (59.3)	50 (40.7)	1.79 (1.13–2.906)	1.50 (1.08–2.75)	
Age category
<30	95 (55.2)	77 (44.8)	1.23 (0.17–8.96)	0.88 (0.10–7.31)	0.9
30–50	45 (44.6)	56 (55.4)	0.80 (0.10–5.93)	0.61 (0.07–5.28)	0.66
>50	2 (50)	2 (50)	1	1	
Suffering chronic diseases
Yes	4 (36.4)	7 (63.6)	0.53 (0.15–1.85)	0.63 (0.12–3.25)	0.58
No	138 (51.9)	128 (48.1)	1	1	
COVID-19 vaccine types
AstraZeneca (reference is No)	108 (53.5)	94 (46.5)	1.38 (0.81–2.36)	1.12 (0.75–2.25)	0.13
Johnson (reference is No)	23 (53.5)	20 (46.5)	0.90 (0.46–1.72)	0.65 (0.35–1.46)	0.22
Sinopharm (reference is No)	0	11 (100)	0.05 (0.01–0.23)	0.03 (0.01–0.18)	0.38
Pfizer (reference is No)	21 (67.7)	10 (32.3)	2.16 (0.98–4.79)	0.88 (0.75–4.23)	0.11
COVID-19 vaccine received
One time	17 (35.4)	31 (64.6)	0.34 (0.12–0.97)	0.24 (0.04–1.30)	0.09
Two times	112 (53.8)	96 (46.2)	0.71 (0.28–1.80)	0.73 (0.20–2.61)	0.63
More than two times	13 (61.9)	8 (38.1)	1	1	
Allergy to foods or medicines
Yes	19 (67.9)	9 (32.1)	2.16 (0.94–4.96)	2.56 (1.86–7.59)	0.04*
No	123 (49.4)	126 (50.6)	1	1	
Substance use
Yes	2 (40)	3 (60)	1.43 (0.23–8.73)	0.98 (0.10–8.94)	0.99
No	139 (51.1)	133 (48.9)	1	1	
Recommend for others
Yes	122 (49.8)	123 (50.2)	0.59 (0.28–1.27)	0.62 (0.27–1.42)	0.26
No	20 (62.5)	12 (37.5)	1	1	

* Indicate significantly associated variables.

## Discussion

During the COVID-19 pandemic, healthcare professionals were among the high-risk populations. Due to their susceptibility in terms of their working conditions, front-line healthcare personnel were given priority when it came to vaccination (3). Evidence of the adverse effects of the COVID-19 vaccines administered in Ethiopia is scarce.

Among the study participants, 51.3% had developed adverse reactions associated with vaccination. The current study’s findings are lower than those of other studies conducted in Ghana, which showed that the prevalence of adverse reactions among study participants was 80.7% (9); in Togo, it was 71.6% (10); and in UAE, it was 64.8% (12). These differences in the prevalence of adverse reactions could be due to variation in sample size and socioeconomic status.

The major adverse effects reported by the COVID-19 vaccine recipients were pain at the site of injection (47%), fatigue and drowsiness (28.2%), and joint/muscle pain (23.1%), followed by headache (17.7%) and fever (14.4%). A survey based on a mobile self-report questionnaire to assess the prevalence and characteristics of adverse reactions following the first dose of the ChAdOx1 nCoV-19 Vaccine and the BNT162b2 vaccine was conducted among healthcare workers in South Korea. Of the 5,589 healthcare workers in the ChAdOx1 nCoV-19 group, the overall adverse reaction rate was 93%. Approximately, half of the ChAdOx1 nCoV-19 group reported moderate or severe grade events (13).

In the current study, only 0.7% had developed adverse reactions that led to hospitalization. Comparable findings were noted in a study conducted in Southern Ethiopia, which showed that 1.1% had severe adverse reactions (14), and in Togo, where 1% were found to have been hospitalized (10). These comparable findings from different studies might implicate the rare occurrence of severe adverse reactions associated with the COVID-19 vaccine.

The most reported short-term adverse reactions were headache (41.2%), fever (37.5%), fatigability and tiredness (33.9%), chills (33.2%), muscle pain (28.5%), and decreased sleep quality (12.3%). These findings are comparable with other studies conducted in Ghana (9), Togo (10), and Southern Ethiopia (14). The similarities could be due to the wide scale use of the AstraZeneca vaccine in this population.

The present study revealed that only 1.4% had been diagnosed with thrombosis (blood clots). This finding contradicts a study conducted in Ethiopia, which showed none of the study participants reported laboratory-confirmed blood clotting problems (11). However, a systematic review and meta-analysis study indicated that venous thrombosis due to the COVID-19 vaccine was 28 per 100,000 doses (15). Similarly, other systematic reviews and exploratory analysis studies indicated the presence of venous thrombosis due to the COVID-19 vaccine (16). According to this systematic review and exploratory analysis study, the pathophysiology behind venous thrombosis is explained as follows: “New experimental studies have assumed that thrombosis is related to a soluble adenoviral protein spike variant, originating from splicing events, which cause important endothelial inflammatory events, and binding to endothelial cells expressing ACE2” (16) (p.2).

Multivariable logistic regression analysis showed that the odds of experiencing adverse reactions were 1.501 times higher among women than men. Similar findings were noted in a study conducted in Togo (10). This result may be explained by a greater immunological response brought on by estrogen (6) or other unidentified immunologic differences between men and women (10).

This study revealed no statistically significant correlation between the different COVID-19 vaccination types and associated adverse reactions. These results suggest that unfavorable reactions to a vaccine are not influenced by the type of vaccine.

## Conclusion

In this study, adverse reactions following the COVID-19 vaccine were moderate in magnitude and minimal in severity. This study revealed that 51.3% of participants had developed adverse reactions associated with vaccination. The majority of the study participants (72.9%) had received the AstraZeneca vaccine. The most reported short-term adverse reactions following vaccination were headache, fever, fatigability and tiredness, chills, and muscle pain. Less than 1% (0.7%) had developed adverse reactions that led to hospitalization. The present study revealed that the occurrence of thrombosis (blood clots) was rare. In the current study, the odds of having adverse reactions were higher among women than men. The type of COVID-19 vaccine had no significant association with adverse reactions.

Based on our findings, we recommend health professionals receive any of the COVID-19 vaccines without fear or hesitancy since severe adverse reactions were found to be rare. Future national, multicenter, prospective, and randomized study should be conducted to assess the independent association of each vaccine with adverse reactions. Our results show that women were more likely to develop adverse reactions than men. Therefore, future randomized control studies should investigate this association clearly.

## Data availability statement

The raw data supporting the conclusions of this article will be made available by the authors, without undue reservation.

## Ethics statement

The study was conducted after obtaining ethical approval letters from the (IRB) of the College of Medicine, Asrat Woldeyes Health Science Campus, Debre Berhan University, and Addis Ababa Health Bureau complied with the Declaration of Helsinki. A permission letter was obtained from the study hospitals. The data were collected after obtaining written informed consent from the study participants. To keep confidentiality, codes were used and unauthorized persons did not have access to the data.

## Author contributions

AA contributed to conception or design, data collection, acquisition, analysis, or interpretation, drafted the manuscript, and critically revised the final manuscript. ND contributed to conception or design and data collection, and drafted the manuscript. AB, FT, and DM contributed to acquisition, analysis, or interpretation, drafted the manuscript, and critically revised the final manuscript. BG drafted the manuscript and critically revised the final manuscript. All authors contributed to the article and approved the submitted version.

## Abbreviations

ADRs: Adverse drug reactions
AEFI: Adverse events following immunization
AEs: Adverse effects
CDC: Center for Disease Control and Prevention
CVST: Cerebral venous sinus thrombosis
EMA: European medicines agency
HCPs: Health care professional
NDVP: National deployment and vaccination plan
WHO: World Health Organization
